# Data Management of Sensitive Human Proteomics Data: Current Practices, Recommendations, and Perspectives for the Future

**DOI:** 10.1016/j.mcpro.2021.100071

**Published:** 2021-03-10

**Authors:** Nuno Bandeira, Eric W. Deutsch, Oliver Kohlbacher, Lennart Martens, Juan Antonio Vizcaíno

**Affiliations:** 1 Center for Computational Mass Spectrometry, University of California, San Diego (UCSD), La Jolla, California, USA; 2 Department Computer Science and Engineering, University of California, San Diego (UCSD), La Jolla, California, USA; 3 Skaggs School of Pharmacy and Pharmaceutical Sciences, University of California, San Diego (UCSD), La Jolla, California, USA; 4 Institute for Systems Biology, Seattle, Washington, USA; 5 Institute for Bioinformatics and Medical Informatics, University of Tübingen, Tübingen, Germany; 6 Quantitative Biology Center, University of Tübingen, Tübingen, Germany; 7 Biomolecular Interactions, Max Planck Institute for Developmental Biology, Tübingen, Germany; 8 Institute for Translational Bioinformatics, University Hospital Tübingen, Tübingen, Germany; 9 VIB-UGent Center for Medical Biotechnology, VIB, Ghent, Belgium; 10 Department of Biomolecular Medicine, Ghent University, Ghent, Belgium; 11 European Molecular Biology Laboratory, European Bioinformatics Institute (EMBL-EBI), Wellcome Trust Genome Campus, Hinxton, Cambridge, United Kingdom

**Keywords:** mass spectrometry, proteomics, databases, ethics, policy, controlled access data, AD, Alzheimer’s disease, CPTAC, Clinical Proteome Tumor Analysis Consortium, DAC, Data Access Committee, dbGAP, database of Genotypes and Phenotypes, DDA, Data-Dependent Acquisition, DIA, Data Independent Acquisition, EBI, European Bioinformatics Institute, EGA, European Genome-phenome Archive, FDR, False Discovery Rate, GA4GH, Global Alliance for Genomics and Health, GDPR, General Data Protection Regulation, GEO, Gene Expression Omnibus, HCD, Higher-energy collisional dissociation, HIPAA, Health Insurance Portability and Accountability, IRB, Institutional Review Board, JGA, Japanese Genotype-phenotype Archive, MS, Mass Spectrometry, NIH, National Institutes of Health, NIST, National Institute of Standards and Technology, PII, Personally Identifiable Information, PRM, Parallel Reaction Monitoring, PSM, Peptide Spectrum Match, PTM, Posttranslational Modification, SAAVs, Single Amino Acid Variants, SMEs, Small and Medium-sized Enterprises, SRM, Selected Reaction Monitoring, VCF, Variant Call Format

## Abstract

Today it is the norm that all relevant proteomics data that support the conclusions in scientific publications are made available in public proteomics data repositories. However, given the increase in the number of clinical proteomics studies, an important emerging topic is the management and dissemination of clinical, and thus potentially sensitive, human proteomics data. Both in the United States and in the European Union, there are legal frameworks protecting the privacy of individuals. Implementing privacy standards for publicly released research data in genomics and transcriptomics has led to processes to control who may access the data, so-called “controlled access” data. In parallel with the technological developments in the field, it is clear that the privacy risks of sharing proteomics data need to be properly assessed and managed. In our view, the proteomics community must be proactive in addressing these issues. Yet a careful balance must be kept. On the one hand, neglecting to address the potential of identifiability in human proteomics data could lead to reputational damage of the field, while on the other hand, erecting barriers to open access to clinical proteomics data will inevitably reduce reuse of proteomics data and could substantially delay critical discoveries in biomedical research. In order to balance these apparently conflicting requirements for data privacy and efficient use and reuse of research efforts through the sharing of clinical proteomics data, development efforts will be needed at different levels including bioinformatics infrastructure, policymaking, and mechanisms of oversight.

## Highlights

• Availability of proteomics data in public repositories (ProteomeXchange) has become the norm.
• There are growing ethical issues and legal requirements related to human clinical proteomics data.
• We review the current state of the art and provide our thoughts about some proteomics data types.
• We make concrete recommendations to address these issues, summarized in four different points.

## Graphical Abstract

**Figure:**
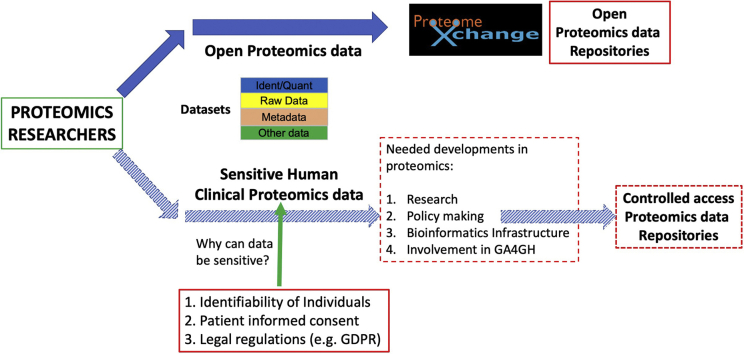

## In Brief

Availability of proteomics data in the public domain has become the norm, as it has been the case in genomics and transcriptomics for many years. Analogously to sequencing data, there are increasing ethical issues and legal requirements related to sensitive human clinical proteomics data. We review the current state of the art and make concrete recommendations to address these issues in the proteomics field, which are summarized in four different areas.

High-throughput proteomics approaches have matured significantly, becoming an increasingly used tool in biological and clinical research. The main high-throughput proteomics approach today is mass spectrometry (MS) coupled to liquid chromatography, with less commonly used proteomics approaches based on antibodies (*e.g.*, protein arrays and other immunofluorescence-based techniques) and protein expression studies using affinity reagents such as aptamers (1). Proteomics often complements information gained from other omics techniques such as genomics and transcriptomics (proteogenomics and proteotranscriptomics), metagenomics (metaproteomics), glycomics, and metabolomics. Multiomics studies involving proteomics approaches are becoming increasingly common, including in the context of personalized medicine. The most high-profile examples are focused on cancer, such as those led by the CPTAC (Clinical Proteome Tumor Analysis Consortium) (2).

In parallel to the many technical developments in the field, open data policies have developed over the last few years as well. This process has largely followed the trends set by neighboring disciplines such as genomics and transcriptomics. As a result, it is now commonplace that all acquired proteomics data that support the conclusions in scientific publications are made available in public proteomics data repositories. One of the main benefits of this public data availability is that it enables experimental reproducibility and an independent assessment of the results described, while also potentially enabling new discoveries as more advanced algorithms become available. Indeed, it enables reuse of public data sets in many different ways, including “big data” approaches such as the development of machine-learning-based predictors of analyte behavior (3) and the extraction of new knowledge (4, 5). This shift toward public data release has been supported by the stricter data availability requirements from scientific journals and funding agencies, which in turn forms part of the wider movement toward open science practices in biology (6, 7).

An important foundation for these pervasive open data policies has been provided by the perceived reliability of public proteomics data resources. While the first of these resources were established around 2004–2005, in 2011, the most prominent ones came together and started to collaborate formally within the ProteomeXchange (PX) consortium (http://www.proteomexchange.org/) (8), resulting in unified standard submission and data dissemination practices. PX has evolved and grown since its founding and is at present composed of six members: the PRIDE database (9) (Hinxton, UK), PeptideAtlas/PASSEL (10) (Seattle, USA), MassIVE (http://massive.ucsd.edu/, San Diego, USA), Panorama Public (Seattle, USA) (11), jPOST (the jPOST project, Japan) (12), and iProX (13) (Beijing, China). Overall, as of February 2021, more than 24,000 PX data sets have been submitted to PX resources and more than 500 new data sets are submitted every month on average (14). Over 45% of the data sets include data coming from human samples (including cell lines).

With an increase in the popularity of clinical proteomics studies, one quickly emerging topic is the management of clinical, potentially sensitive, human proteomics data (15). A key question in this topic is whether proteomics data from human samples threaten the privacy of the human donors and/or that of their family members (16). And if so, what is the severity and likelihood of this potential privacy risk? The introduction of the General Data Protection Regulation (GDPR) by the European Union was an important trigger for this topic, and recent publications have highlighted the importance of considering the GDPR in clinical proteomics (17) as well as in public bioinformatics resources (18). In the United States, several legal and ethical frameworks exist that address data privacy including The Privacy Act of 1974 (19), the Health Insurance Portability and Accountability Act of 1996 (HIPAA) (20), the 2013 HIPAA Privacy Rule Amendment (21), and the Code of Federal Regulations 45 part 46 (the Revised Common Rule) (22).

Implementing privacy standards for publicly released research data has led to processes to control who may access the data, so-called “controlled access” data. For data with potentially identifying information, such as germline genomic sequence data, there is a mechanism whereby potential data users must first be approved by a Data Access Committee (DAC). This DAC confirms that the applicant is a *bona fide* researcher from a recognized research institution and that the proposed data use is allowable under the data use limitations of the data set. Resources that support the storage and dissemination of such controlled access data have already been developed and are commonly used for DNA and RNA sequences, including the European Genome-phenome Archive (EGA, https://www.ebi.ac.uk/ega/), the database of Genotypes and Phenotypes (dbGAP) (23), and the Japanese Genotype-phenotype Archive (JGA) (24). While controlled access data are not freely available to all interested users, ideally, those researchers who can genuinely benefit from access to these data will be provided with access. Researchers who cannot demonstrate a justifiable need to access these data will, however, be denied access. Yet even researchers who are given access need to abide by preset rules regarding these data, which typically entail that the researcher is prohibited from releasing or redistributing these data and often is required to delete these data again after a set period of time. This system of controlled access is designed to enable legitimate use of privacy-sensitive data, while reducing as much as possible the risk of nefarious use. In this context, the GA4GH (Global Alliance for Genomics and Health, https://www.ga4gh.org/) Data Security workstream has defined a set of control objectives, which form the basis of the principles for managing controlled access data (https://www.ga4gh.org/genomic-data-toolkit/data-security-toolkit/).

The onus for privacy protection of human subjects in research rests on the institution conducting the research. As a concrete example of the current state of data management practices, in the United States, according to the 2015 NIH (National Institutes of Health) Genomic Data Sharing Policy, the research institution must provide an assurance to the NIH that an Institutional Review Board (IRB) conducted an ethics review of the proposed study to verify that (see https://grants.nih.gov/grants/guide/notice-files/not-od-07-088.html):

• The data submission (was) consistent, as appropriate, with applicable, national, tribal, and state laws and regulations as well as relevant institutional policies.
• Any limitation on the research use of the data was expressed in the informed consent documents.
• The identities of research participants (would) not be disclosed through the NIH designated data repositories.
• An IRB, and/or Privacy Board, and/or equivalent body, as application, (had) reviewed the investigator’s proposal for data submission and assure(d) that:
  • The protocol for the collection of genomic and phenotypic data is consistent with 45 CFR Part 46.
  • Data submission and subsequent data sharing for research purposes (we)re not inconsistent with the informed consent of study participants from whom the data were obtained.
  • Consideration was given to risks to individual participants and their families associated with data submitted to NIH-designated repositories and subsequent sharing.
  • To the extent relevant and possible, consideration was given to risks to groups or populations associated with submitting data to NIH-designated data repositories and subsequent sharing.
  • The investigator’s plan for deidentifying data sets is consistent with the standard outline in the Policy.

As proteomics techniques become more mainstream and technological developments enable the detection of larger proportions of the proteome with higher coverage, including the reliable detection of single amino acid variants (SAAVs), it is clear that privacy risks of proteomics data can emerge and that these will need to be properly assessed and managed. The proteomics community therefore needs to develop rules and best-practice guidelines to deal with such privacy-sensitive data sets and moreover, needs to evaluate the alignment of these proteomics efforts with sequencing data coming from genomic and transcriptomic approaches.

Currently, data collected as part of biological research are required to be controlled access when: (1) the data contains protected patient information or otherwise could potentially uniquely match to a single individual; (2) the informed consent forms specified that the research data would be controlled access; and/or (3) it is required for adherence to laws and/or regulations concerning data privacy for the citizens (*e.g.*, under GDPR in the EU). Do any of these three scenarios currently apply to proteomics data? Generally, in the United States, IRBs have concluded that proteomic studies do not involve human subjects because the resulting proteomic data are not considered identifiable. This allows proteomic data to be shared freely and openly. However, if an IRB deemed proteomic data identifiable, then the resulting data would be subject to any data use limitations as defined in the informed consent form for that study. That is, the data sharing plan should align with the data uses to which the human subjects agreed, which in some cases may include agreement with public access to the shared data (see https://grants.nih.gov/grants/guide/notice-files/not-od-07-088.html).

We here summarize the conclusions that have emerged on this topic from two meetings held in 2019, plus some follow-on discussions, with a main focus on data management practices. Additionally, we make concrete recommendations about future needed developments. In our view, the community must be proactive in addressing these issues. On the one hand, avoiding addressing the potential of identifiability in proteomic data could in our view lead to reputational damage of human donors and of the field as a whole. On the other hand, making open access to public data more difficult will inevitably reduce access and reuse of proteomic data, thus slowing advances in biomedical research. A balanced approach is therefore necessary.

## Meetings

The conclusions highlighted in this article are derived from extensive discussions at two meetings held in 2019, plus some follow-on discussions. The first meeting was held on April 24th in Amsterdam (The Netherlands), funded by ELIXIR, the European infrastructure for life science data (https://elixir-europe.org/). A second follow-up meeting took place during the Computational Proteomics Seminar in Schloss Dagstuhl (Germany) on August 25th–30th. Overall, there were more than 20 attendees in total to both meetings, representing different stakeholders in the fields of proteomics, bioinformatics, and genomics, including academics and representatives from SMEs (Small and Medium-sized Enterprises) and industry. The complete list of the attendees has been included in the Acknowledgements.

## Do Proteomics Data Constitute Personally Identifiable Information (PII)?

The US law, HIPPA, denotes 18 categories of information as “identifiers” (20). While protein sequences (or genomic sequences) are not explicitly named as identifiers, the list does include “biometric identifiers such as retinal scan or fingerprints, or any other unique identifying number or code” (https://www.hipaajournal.com/considered-phi-hipaa/). Should proteomic data be considered as a HIPAA identifier?

First, consider genomic data as an HIPAA identifier. DNA variants have been incorporated in multiple industrial processes. The forensic industry currently uses DNA as evidence to support a suspect’s presence at the scene of a crime. Genomic information is also used to confirm parental identity and to connect distant relatives through a common ancestor. It can also reveal the presence of genetic diseases and individual disease risks. On the research side, Homer *et al.* demonstrated a successful attack in genome-wide association studies (GWAS), where only aggregated genotype data were openly available and individual genotypes were controlled access (25). The attack was to prove that an individual was a member of the aggregated data set. As a result of this publication, the NIH moved GWAS genotype data to controlled access (26). Another relevant example is the study where Gymrek *et al.* used short tandem repeats on the Y-chromosome from the HapMap project in conjunction with public genetic genealogy databases to recover surnames of research participants (27). Clearly, research publications as well as commercially available industrial applications demonstrate the possibility of identification of an individual based on genomic information.

While the sequence reads from DNA-seq and RNA-seq are usually 75+ nucleotides long, determined *de novo*, and generally acquired with sufficient overlapping read coverage such that error rates in variant calling can easily be quite low, peptide spectrum matches (PSMs) from MS/MS spectra are usually matched against possible answers from a preexisting sequence database and generally have low protein sequence coverage. Contrary to the DNA/RNA sequencing raw data, the typical MS/MS spectrum cannot be interpreted *de novo* to give complete peptide sequence, because fragmentation along on the peptide backbone during MS/MS is usually partial. As a consequence, a key difference between genomic and proteomic data in terms of identifiability is the number of observed variants. A 2016 study of 105 breast cancer tumors showed 80,093 germline DNA variants, 49,986 RNA variants, and only 3620 protein variants (28). Multiple reasons for this disparity among the detected variants between nucleotide sequencing and proteomic data can be identified. First, many DNA variants occur in the intronic regions, which are not translated to protein. Second, some genomic variants may disrupt the translation process such that the variant protein is not expressed. Additionally, at any time—and dependent on the tissue—there will only be at best a bit more than half of the genes that are detected to be translated to a significant degree. Third, there is some redundancy involved in RNA sequence translation to amino acid sequence due to some amino acids being encoded by more than one codon. Fourth, genomic and proteomic data differ in their extent of sequence coverage. Whole-genome sequencing reads each nucleic acid base multiple times leading to sequence coverage of greater than 99%. In proteomics, a protein is often identified by two or even just one representative peptide, resulting in a low sequence coverage. This huge difference in coverage is due to several reasons. Primarily, typical proteomics workflows measure 8–35 residue tryptic peptides, the vast majority of which contains a C-terminal arginine or lysine. Despite the high prevalence of these residues (lysine and arginine each make up 5% of amino acids on average in human proteins), some protein regions contain stretches of hundreds of residues without arginine or lysine. Such regions are not amenable to tryptic digestion and are undetectable by common proteomics workflows. At the time of writing, the NIST (National Institute of Standards and Technology) human HCD (Higher-energy collisional dissociation) TMT peptide spectral library, created by combining dozens of different human samples, contains spectra for 386,224 distinct peptide sequences, which account for a 29.1% proteome sequence coverage (https://chemdata.nist.gov/dokuwiki/doku.php?id=peptidew:lib:human_hcd_tmt). In another ongoing effort, the resource MassIVE-KB covers 50% of the human proteome with spectra for >2.1 million distinct precursors (5). As a conclusion, this leaves a significant portion of the proteome undetected, including any potentially identifying variants that occur in the undetected portion.

However, it should be noted that unlike in genomics and transcriptomics, research into whether proteomics data is actually PII or not remains scarce (29, 30, 31, 32, 33). Nevertheless, ongoing efforts are touching upon this issue, such as the “Proteos” program, which has a forensic angle (https://www.iarpa.gov/index.php/165-research/current-research/cause/proposers-day/979-proteos).

Identification of individuals is very difficult without the detection of rare variants. The exact number of variants required depends on the frequency of the alternative alleles. Indeed, if an allele is very rare, then it can dramatically reduce the number of people identifiable with that SAAV, and one would only need a handful of these to reduce the size of a given pool of samples to just one individual. On the other hand, if an alternative allele is very common, then one would need more of these to make a unique match (29). In the case of tumor samples (*e.g.*, in the case of the CPTAC datasets), the question about identifiability becomes more difficult to answer, due to the fact that, for different tumors, the rate of mutation is very different. For some of them, there is not that much variation between primary and metastases. However, others mutate rapidly and have rapidly diverging somatic signatures (34). Moreover, for either normal or tumor tissue, the calculations about the number of variants required for identifying an individual need to be balanced with the FDR (False Discovery Rate) calculations derived from the proteomics analysis. Overall, the “true” identification of an individual remains very difficult at present, but it is much more plausible to match two data sets to each other or to be able to match a proteomics data set to a genomic/transcriptomics data set, based on a set of identified SAAVs.

In addition to SAAVs, it has been recently reported that individuals could potentially be identified by their protein expression levels in blood plasma (33). To demonstrate this, the authors used a plasma proteomics weight loss study in which samples of 42 individuals were obtained at different time points over 1 year. The reason behind the identifiability risk was twofold: (i) intraindividual correlations of protein expression profiles over time were much higher than the correlations obtained at any point in time between two different individuals; and (ii) highly individual-specific proteins can have more than 100-fold different concentrations between different individuals, but are very constant over time. One example of such proteins is the apolipoprotein (a).

We would therefore like to encourage the community to design and implement further studies to obtain more extensive scientific evidence on the identifiability of proteomics data. This should be done for the main proteomics data types and different types of proteomics workflows and will be an essential component of future, adequately informed policy-related decisions.

## Current Data Dissemination Practices for Proteomics Data Sets

At present, all PX resources are committed to completely open data, which means that there currently are no limitations for data reuse by the community. All PX resources are currently also moving toward formalizing an (at least) default CC-0 license, which is equivalent to publishing into the public domain (the exception is Panorama Public, which implements a CC-BY license, requiring attribution when data is reused). Outside the mainstream PX resources, there are already proteomics datasets for which controlled access has been set up, *e.g.*, a large Alzheimer’s disease (AD) data set described in (35), which has been made available in the generalist data sharing platform AD knowledge Portal (https://adknowledgeportal.synapse.org/). In contrast, proteomics data from the proteogenomic data studies of the CPTAC consortium are openly available at the Proteomic Data Commons (https://pdc.cancer.gov/pdc/), while the corresponding genomic sequence data are available through controlled access at the Genomic Data Commons (https://portal.gdc.cancer.gov/).

## Recommendations for Developing Policy for Proteomics Data Dissemination

### Guiding Principles

As the overall guiding principle, we believe that data management practices for sensitive clinical proteomics data sets must never be more restrictive that the current state of the art for transcriptomics RNA-Seq data. The current situation for gene-expression information is that a large amount of human data sets are made publicly available without restrictions *via* resources such as GEO (Gene Expression Omnibus) (36), ArrayExpress (37) and the Genomic Expression Archive (38). Only where required, sensitive human clinical gene expression data (as decided by the corresponding DACs) is made available in controlled access mode, in appropriate resources such as EGA, dbGAP, or JGA. In addition to gene expression data archives, there are bioinformatics resources that consistently reanalyze data sets and make the results available openly to the public, such as the Expression Atlas (39) at the European Bioinformatics Institute (EBI).

An analogous scenario would be, in our opinion, perfectly transferable to the proteomics field. This model could be summarized with the principle “as open as possible, as closed as necessary.” This way, two main goals would be achieved: ensure that open policies in proteomics are not hindered (and the corresponding benefits for the field remain) while, at the same time, ensuring that potential risks for individuals are appropriately considered and managed where required. Also, it is important to highlight that, as it already happens in transcriptomics, many of the risks associated with sensitive clinical data sets could be managed by making “aggregated” data available to the community. Below, we provide our concrete recommendations concerning different data types and different workflows.

### Recommendations for Different Proteomics Data Types

We believe that the various data types included in a typical proteomics experiment have a different potential for being PII and thus different requirements for being labeled as controlled access information. These recommendations are intended to be applicable to the main data types coming from human samples, including also metaproteomics data, as detailed below.

(a) Raw data (MS data derived directly from the mass spectrometers). These have the highest information content and come with the highest potential for detailed reanalysis using different bioinformatics approaches, potentially even several years after they were initially generated. Therefore, in our view, these raw data have the highest potential to be PII. A possible approach to make this data type less risky would be to provide a “de-identified” version (*e.g.*, by removing a subset of the mass spectra), but it is not clear to us at present how this could be achieved in an efficient way, without removing the vast majority of the spectra. Another possible strategy, at least in the case of label-free quantitative approaches, would be to pool the data from multiple samples in each cohort, but this would reduce the statistical power of the analysis, and it is not clear how many samples would need to be pooled to effectively reduce the risk of identifiability to acceptable levels. This would not apply in the same manner to labeling approaches (*e.g.*, TMT), as the reporter ions would retain the individual information about each sample. In the absence of any effective anonymization strategy at present, sensitive raw data coming from human studies should be considered potentially PII and managed through controlled access mechanisms. As mentioned above, different ameliorating strategies (*e.g.*, by removing a subset of the spectra, data pooling) could be developed to avoid this.
(b) Search databases (FASTA or PEFF files (40)). Sample-specific protein sequences should only be made available if these do not include any SAAV-related information. Customized search databases containing individual human sequences should be considered PII data and managed through controlled access mechanisms. This is already common practice in the CPTAC data portal. However, in our view, aggregated SAAV containing protein sequences derived from an entire patient cohort should not be considered PII.
(c) Identified peptide and protein sequences. If a generic protein sequence database (*e.g.*, UniProtKB (41) reference proteome) is used for the analysis, they should not pose any risk. However, if personal proteomes including SAAVs have been used, specific sequence variants could be potentially identified, conferring the potential to these data types to be PII. However, it would be straightforward from the results to remove any sequences corresponding to variants that could lead to individual identifiability allowing the PII risk to be mitigated for these data. An intermediate approach would be to include in the search database only those sequence variants that are present over some suitable threshold of allele frequency in the population, because, as mentioned above, rare alleles are much more critical for the identification of individuals. In any case, peptide and protein sequences are less risky than raw data and, if these were obtained through matching to reference protein sequences (*e.g.*, the UniProtKB reference proteome, with the potential inclusion of only very common variants), they should not be considered PII.
(d) Peptide and protein expression values. These data types are, in our view, analogous to gene expression values, so the same principles followed in transcriptomics should be applied. To address the identifiability risks related to individual protein levels, peptide and protein expression values could therefore be aggregated and averaged to be made openly available. Additionally, for the same reason explained above, expression values should be reported using only canonical protein sequences as reference system, with the potential inclusion of only very common variants to mitigate PII risks. However, in the case of data aggregation (*e.g.*, reporting SAAV frequencies or distributions of their abundances in each cohort of patients), it should be required that the study contains a minimum number of samples, which is something that would need to be investigated appropriately.
(e) Expression profiles of modified peptides/proteins. The principles used for peptide/protein expression values apply equally to peptides/proteins with posttranslational modifications (PTMs). Reporting of identifications modified with biological, chemical, or artifactual modifications should not represent any issues, except possibly in cases where there is ambiguity in whether a modification mass (or combination of masses) could also correspond to the mass difference of an SAAV. In the concrete case of open modification searches, if this is the used analysis method, unexplained delta masses for putative PTMs should not be made openly available since they could represent (combinations of) SAAVs.

### Recommendations for Different Proteomics Approaches

The main proteomics approaches used in “discovery” mode are Data-Dependent Acquisition (DDA), Data-Independent Acquisition (DIA), and top-down proteomics. In addition, there are targeted approaches such as Selected Reaction Monitoring (SRM) and Parallel Reaction Monitoring (PRM). Do these techniques have different potential to be PII? In our view, although the current state of the art is different for the different techniques, no differences in terms of potential risks should be considered for “discovery” approaches as a whole.

Targeted approaches are however different, and there could be different scenarios depending on each specific study. For instance, it is obvious that if specific variant peptides were targeted in a given study (especially those that are rare), analyses would pose a higher risk of being PII than analogous targeted analyses performed on nonvariant peptides.

### Experimental Metadata

The metadata information currently available for each originally submitted PX data set is defined to describe only high-level experimental information and does not contain a detailed description of samples, study groups, or patient characteristics. Therefore, current metadata in PX resources does not pose any risk to be PII. In case more detailed metadata or specific sample/clinical information would be submitted, this information would need to be deidentified first or should be made available as controlled access data. This is already a common practice in DNA/RNA sequencing studies, and the same analogous recommendations would therefore apply to similar metadata for proteomic studies. However, it should be noted that, at least at present, there is little standardization in the sample/clinical metadata provision for clinical sensitive human studies. In fact, different criteria exist *e.g.*, in different countries, consortia, etc. As an illustrative example, the metadata requirements for the EGA can be accessed at https://www.ebi.ac.uk/ega/submission/sequence/metadata.

### Reanalyses of Human Data Sets and Spectral Libraries

Reanalyses of public proteomics data sets are becoming increasingly common. For instance, PX resources (*e.g.*, PeptideAtlas and MassIVE) are starting to track reanalyzed data sets, by using RPXD identifiers for data sets (14), instead of the PXD identifiers used for originally submitted data sets. If controlled access is implemented for the relevant data sets, these bioinformatics resources would also need to apply to DACs to gain access to data sets prior to reanalysis. Such an application should contain the actual analysis protocol that will be performed, as well as the way in which any results will be presented to the scientific community.

The prescription offered above for peptide/protein expression values would apply here as well, meaning that final results should be made available using only canonical proteins as the reference system, containing no SAAV information. Data coming from different individuals should also be aggregated and averaged prior to being made openly available. This process would again be analogous to what happens in transcriptomics resources that perform reanalysis of RNA-Seq data sets, such as EBI’s Expression Atlas.

One particular use case for data reanalysis and reuse is the creation of spectral libraries. As indicated above, raw data has the highest potential to be PII. The objective to create spectral libraries coming from controlled access data sets should therefore be included in applications to DACs and should always be done only after aggregating into a large-enough collection of spectra (ideally from multiple data sets).

## Concrete Recommendations for Performing Data Submissions

Authors that are required by their DACs or legal advisors to classify proteomics data as PII are advised to find *alternative ways* to make the data available for the community as PX resources at present cannot provide controlled access to data sets as EGA, dbGAP, and/or JGA do for DNA/RNA sequencing data. This raises two questions:

(1) Are there alternative resources at present? To the best of our knowledge, proteomics data are not supported by EGA, dbGAP, and/or JGA at present because these resources are focused in nucleotide sequencing experiments. Institutional repositories (*e.g.*, those available in some universities and/or research institutions) may offer a local alternative for some researchers. Additionally, as it was mentioned above, the collaborative data sharing platform AD knowledge Portal already contains one controlled access proteomics data set. Yet, it is likely that a significant proportion of the generated clinical proteomics data are currently simply not being submitted to any public resource at all, although it is very difficult to estimate this number. As a result, the paradoxical situation is that the availability of relevant infrastructure for controlled access sharing of proteomics data with substantial PII risk may well increase the availability of these data rather than reduce it.
(2) Are proteomics scientists requesting this functionality currently? The vast majority of the field is currently not worried about these potential issues. In particular, the CPTAC program is by far the largest proteomics study of human subjects to date and, as also mentioned above, proteomics data has been classified by the NIH IRB at not PII—a precedent that should be considered by US institutions deciding how to classify their own proteomics data. On an international scale, PRIDE, the most-used proteomics data repository, storing approximately 5300 data sets during 2020 alone, received just a handful of queries around this topic over the last couple of years. Only one of these queries resulted in triggering the deletion of a data set that had already been submitted, although it had not been publicly released.

## Future Needed Developments: What Is the Way Forward?

The whole field will undoubtedly need to transition to take potential ethical and privacy issues formally into account. Otherwise, there is a real risk of reputational damage and potential negative consequences for patients involved in clinical proteomics studies. It can be expected that standard data management and dissemination practices will evolve in parallel, as awareness about these issues grows. However, it is not unrealistic to anticipate that some sensitive proteomics data sets will formally need to be made available in controlled access mode, so it is also safe to posit that alternative data submission and dissemination mechanisms will have to be developed by public resources to support those use cases. In fact, it would be vastly preferable that the necessary infrastructure is available before the PII issue in proteomics comes to a head, as this will allow the field to transition much more smoothly toward a greater availability of proteomics data with a substantial PII risk. Therefore, in our view, developments are needed at different levels:

(1) Research. As mentioned above, it is important that larger-scale studies be performed to learn more about the identifiability risks for the different proteomics data types and approaches, to allow well-informed decisions in the future.
(2) Policymaking. Specialists in biological data policy need to understand the different data types included in proteomics experiments and the inherent differences with DNA/RNA sequencing data. Specifically, assigning certain human proteomic data as PII has implications for obtaining informed consent from human donors as investigators requesting human samples for proteomic studies will then need to ensure the samples have been consented for generating potentially identifying information through the research. Members of the DACs must also receive adequate training with the same overall objective in mind.
(3) Bioinformatics Infrastructure. The necessary infrastructure (data repositories, submission pipelines, interfaces to access controlled data, which are compliant with the existing practices today for DNA/RNA sequencing data, and possibly tailored data formats) needs to be put in place so that controlled access proteomics data can be adequately supported. Funding agencies must also realize that a substantial investment will be required to create and support this whole ecosystem.
(4) Involvement of proteomics groups in activities of the GA4GH, in order to learn how the genomics community is already handling these types of issues, at both the technical (infrastructure) and policy levels. As a concrete example, one possibility could be to adapt the approach of the existing Beacons framework, initially developed for DNA sequence variant information (42), to also support variant-level information at the proteome level. As it stands today, proteomics variation information can already be represented in the current version of the Beacons framework, using translated VCF (Variant Call Format) files. There is thus a possibility that the Beacons technical infrastructure could inform how proteomics resources could be extended with this aim, even if it would not solve all possible identification risks (43).

## Conclusions

The right balance needs to be found in providing adequate protections for PII while still allowing for the immense potential benefits of sharing clinical proteomics data. In the most extreme scenario, a proposal in which most human-sensitive proteomics data sets should be treated as controlled access data would undermine the years of work that have gone into creating a culture of open data sharing in the proteomics community. The concomitant loss of data sharing would drastically hinder progress in proteomics and especially its utility in biomedical applications. On the other extreme, continuing with a fully open model for proteomic data sharing will eventually lead to privacy issues for sample donors. A high profile demonstrated breach of privacy would lead to a severe backlash in data sharing policy and cause a substantial erosion of public trust in proteomics research.

We have here proposed a *via media* between these extremes by adhering to the principle “as open as possible, as closed as necessary.” It should be noted that we do not profess to have a full and complete solution for these issues. However, it should be clear that there are three main stages of evolution that should take place in the near future. First of all, a clear policy needs to be developed. Second, additional infrastructure for controlled data sharing will need to be developed for proteomics data, which will in turn require dedicated funds to be raised. Third, the resulting policy will need to be adopted by the community and practiced through the developed infrastructure. Throughout, it should be noted that investments in a clear policy on, and a suitable dissemination infrastructure for, the subset of (clinical) proteomics data at risk of PII will actually enable the sharing of these data, which are otherwise likely to remain locked away, in turn bringing the many benefits of such sharing to clinical proteomics efforts.

We hope that this article can be used to document the current state of the art and that it represents a first step for the community to handle PII-related issues appropriately.

## Conflict of interest

The authors declare no competing interests.
